# Genome-wide analysis of root hair-preferential genes in rice

**DOI:** 10.1186/s12284-018-0241-2

**Published:** 2018-08-29

**Authors:** Sunok Moon, Anil Kumar Nalini Chandran, Gynheung An, Chanhui Lee, Ki-Hong Jung

**Affiliations:** 1 Department of Genetic Engineering and Crop Biotech Institute, Kyung Hee University, Yongin, 17104 Korea; 2 Department of Plant and Environmental New Resources, Kyung Hee University, Yongin, 17104 Korea

**Keywords:** Root hairs, Transcriptome, GUS reporter system, Rice, Comparative analysis, Network analysis

## Abstract

**Background:**

Root hairs are valuable in taking up nutrients and water from the rhizosphere and serving as sites of interactions with soil microorganisms. By increasing the external surface area of the roots or interacting with rhizobacteria, root hairs directly and indirectly promote plant growth and yield. Transcriptome data can be used to understand root-hair development in rice.

**Result:**

We performed Agilent 44 K microarray experiments with enriched root-hair samples and identified 409 root hair-preferential genes in rice. The expression patterns of six genes were confirmed using a *GUS* reporter system and quantitative RT-PCR analysis. Gene Ontology (GO) analysis demonstrated that 13 GO terms, including oxygen transport and cell wall generation, were highly over-represented in those genes. Although comparative analysis between rice and *Arabidopsis* revealed a large proportion of orthologous pairs, their spatial expression patterns were not conserved. To investigate the molecular network associated with root hair-preferential genes in rice, we analyzed the PPI network as well as coexpression data. Subsequently, we developed a refined network consisting of 24 interactions between 10 genes and 18 of their interactors.

**Conclusion:**

Identification of root hair-preferential genes and in depth analysis of those genes will be a useful reference to accelerate the understanding of root-hair development in rice.

**Electronic supplementary material:**

The online version of this article (10.1186/s12284-018-0241-2) contains supplementary material, which is available to authorized users.

## Background

Root hairs are tip-growing extensions that arise from specialized epidermal cells (Leavitt 1904). They have pivotal roles in taking up nutrients and water from the rhizosphere and serve as sites of interactions with soil microorganisms (Dazzo et al. 1984; Gilroy and Jones 2000). By increasing the external surface area of the roots or interacting with rhizobacteria, root hairs directly and indirectly promote plant growth and yield (Curl and Truelove 1986; Bowen and Rovira 1999; Mukerji et al. 2006; Hayat et al. 2010). Therefore, longer and more abundant root hairs are a desirable trait in modern crop breeding programs (Brown et al. 2013).

These morphological characteristics are controlled by environmental and genetic factors (Bates and Lynch 2000; Ma et al. 2001b; Muller and Schmidt 2004; Datta et al. 2011). *Arabidopsis* plants produce dense, elongated root hairs in response to phosphate starvation. In root-hair mutants, phosphate uptake is defective under low-phosphorus conditions (Bates and Lynch 2000). Similar results have been reported for a barley (*Hordeum vulgare*) root-hairless mutant that, under low-phosphorus soil conditions, stops growing and eventually dies after 30 d, thereby indicating the importance of root hairs in phosphate acquisition (Gahoonia et al. 2001; Gahoonia and Nielsen 2003). To demonstrate the effectiveness of these structures in absorbing water from the soil, Carminati et al. (2017) have compared the relationship between the transpiration rate and xylem suction in two different genotypes of barley and found that the root-hairless mutant takes up substantially less water than the wild type (WT) from dry soil. In rice (*Oryza sativa*), the formation and elongation of root hairs is enhanced under aerobic conditions but impaired in flooded soils (Kawata and Ishihara 1959; Kawata et al. 1964). Nitrogen or potassium deprivations also stimulate this elongation, and several genes related to uptake of those nutrients are preferentially expressed in the root hairs (Bhat et al. 1979; Lauter et al. 1996; Hartje et al. 2000; Bregante et al. 2008; Jung et al. 2009). Such elongation is defective in the mutants of *Tiny Root Hair 1* (*TRH1*) and *Arabidopsis K transporter* (*AKT1*), two genes involved in potassium uptake (Desbrosses et al. 2003). Although root hairs are altered under an iron deficiency, the uptake of some nutrients, including iron and silicon, is independent of root-hair status (Moog et al. 1995; Ma et al. 2001a; Muller and Schmidt 2004).

The length and number of root hairs are regulated by genetic factors. Their formation follows one of three patterns: 1) *random*, any cell of the epidermis being capable of root-hair development; 2) *alternative*, morphologically different cells alternating along longitudinal epidermal cell files, with asymmetric cell division creating a pattern of shorter hairs and longer non-hair cells; or 3) *striped*, root hairs and non-hair cells occurring in separate longitudinal files (Dolan and Costa 2001; Datta et al. 2011; Marzec et al. 2014). Root hairs form in epidermal cells that overlie the junction of two cortical cell files (H-position), but not in epidermal cells overlying single cortical cells (N-position). In rice, the patterning of H and N cells along files in the root epidermis is random (Kim and Dolan 2011). Although hair cells are shorter than non-hair cells at maturity, they are morphologically identical when both are first initiated (Kim and Dolan 2011). However, the mechanisms for fate determination and asymmetric epidermal cell elongation have not yet been uncovered.

In *Arabidopsis*, more than 130 genes contribute to the process of root-hair formation (Kwasniewski et al. 2013b). High-throughput analysis, including comprehensive transcriptional profiling of several cell-fate mutants, and ‘omics’ analysis of transcripts and proteins in the root hairs, have provided more information about the development and function of those structures in *Arabidopsis* (Bruex et al. 2012; Lan et al. 2013). Because root hairs are primary sites for the symbiosis of rhizobia in legume species, this has become an important area of research (Brechenmacher et al. 2010). To understand the function of root hairs during rhizobial infections, intensive ‘omics’ studies, e.g., transcriptome, proteome, phosphoproteome, metabolome, and glycolipidome, have been performed with soybean (*Glycine max*) (Brechenmacher et al. 2010, 2012; Libault et al. 2010; Nguyen et al. 2012; Wei et al. 2016). The barley transcriptome and the proteome for *Zea mays* have also been examined (Kwasniewski et al. 2010; Janiak et al. 2012).

Although rice is a major food crop, little research has focused on its formation of root hairs. Several genes, e.g., *OsCSLD1* (*cellulose synthase-like D1*), *EXPA17* (*expansin A*), *RTH1* (*apyrase*), *SRH2* (*xyloglucan 6-xylosyltransferase*), and *OsRHL1* (*bHLH TF*), appear to regulate this development, as demonstrated by a defect in root-hair elongation in their respective mutants (Kim et al. 2007; Ding et al. 2009; Yuo et al. 2009; Yu et al. 2011; Wang et al. 2014). Large-scale, comparative analyses of genes for root-hair development have been conducted with diverse species of vascular plants (Huang et al. 2017). However, no studies have involved global identification and bioinformatic analyses of genes that participate in root-hair development in rice. This lack has limited the possibilities for functional genomic investigation of those structures in this model plant system. Here, we used root hair-enriched samples and Agilent 44 K microarray experiments to identify 409 genes that are preferentially expressed in rice root hairs. We also performed integrative in silico analysis to reveal the functions of these genes.

## Results

### Transcriptome analysis of root hairs to identify preferentially expressed genes

**Fig. 1:**
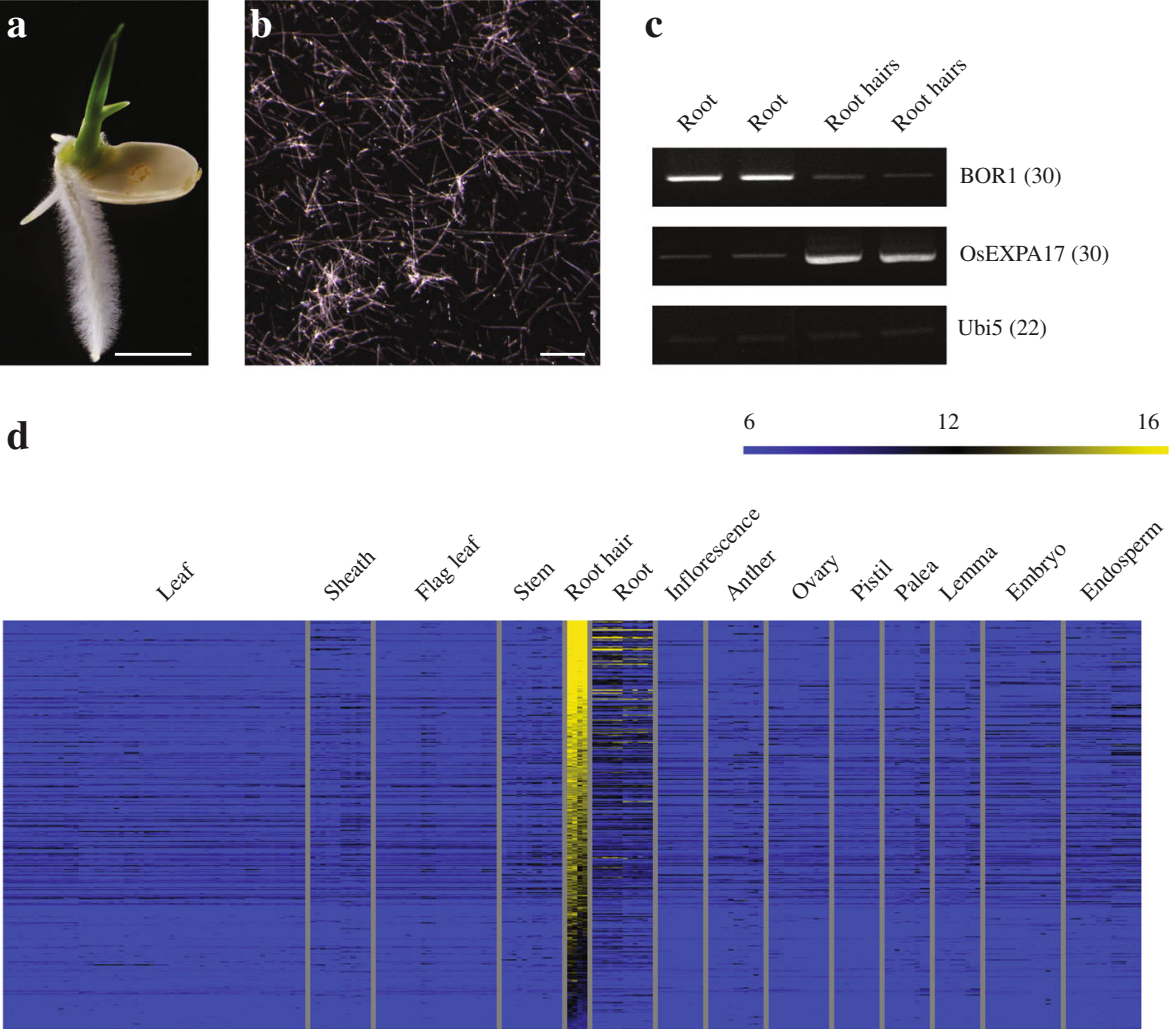
Identification of genes preferentially expressed in rice root hairs. **a** Seedling at 3 DAG, prior to sample preparation. Scale bar = 5 mm. **b** Microscopic observation of root hairs. Scale bar = 0.1 μm. **c** RT-PCR analysis of samples using 2 marker genes: *OsBOR1*, negative marker showing root-preferential expression; and *OsEXPA17*, positive control showing root hair-specific expression. Value in parentheses indicates number of PCR cycles. **d** Heatmap analysis of 409 genes preferentially expressed in root hairs. Yellow, high expression; dark-blue, low expression

For transcriptome analysis of the root hairs, it is critical to collect uncontaminated samples. Because lateral roots protrude from the seminal roots and easily fall off during the purification step, we selected plants at 3 days after germination (DAG), a stage at which the initial emergence of lateral roots was not apparent under our particular growth conditions (Fig. 1a). After brushing the frozen, excised seminal root segments, we collected their long and narrow protrusions (root hairs) and examined them under a microscope. These observations indicated that the samples were highly enriched with root hairs (Fig. 1b). To check the sample status, we performed reverse transcription-PCR (RT-PCR). As the negative control, we used *OsBOR1*, a boron transporter that, under normal conditions, is obviously expressed in the stele, endodermis, and exodermis of roots, but is not expressed in the root hairs (Nakagawa et al. 2007). This gene was highly expressed in our root samples, but only weakly detected in the hair samples (Fig. 1c). We also monitored the expression of *OsEXPA17*, a marker gene for rice root hairs (Yu et al. 2011) and found that it was strongly amplified in the root hairs, but only weakly detected in the roots (Fig. 1c). This indicated that our samples were suitable for microarray analysis to identify genes specifically involved in root-hair development in rice.

The root-hair transcriptome was analyzed using an Agilent 44 K rice genome microarray (GSE109811). To compare the expression profiles with those in other tissues/organs, we downloaded a publicly available anatomical Agilent rice microarray dataset, GSE21494, from the National Center for Biotechnology Information Gene Expression Omnibus (NCBI GEO; http://www.ncbi.nlm.nih.gov/geo/) (Sato et al. 2011). Intensity values for the Agilent array data were initially normalized and log2-transformed. First, we selected 13,929 genes by filtering log2 expression values > 8. We then performed K-means clustering (KMC) analysis using the Euclidean distance metric to group the genes into 12 clusters based on their expression patterns (Additional file 1: Figure S1). Genes that were preferentially expressed in the root hairs were assigned to Cluster 7. Within that cluster, we eliminated some genes that were also highly expressed in other tissues. Although root hairs can account for up to 70% of the total surface area of a root, their proportion is very small in *Arabidopsis*, with just 1% of the protoplasts originating from the hairs of five-day-old seedling roots (Jungk 2001; Lan et al. 2013). Therefore, we selected genes for which expression was at least two-fold higher in the root hairs than in the roots. Finally, we identified 409 genes preferentially expressed in root hairs (Fig. 1d; Additional file 2: Table S1).

### Confirmation of expression patterns for genes preferentially expressed in root hairs via the *GUS* reporter system

**Fig. 2:**
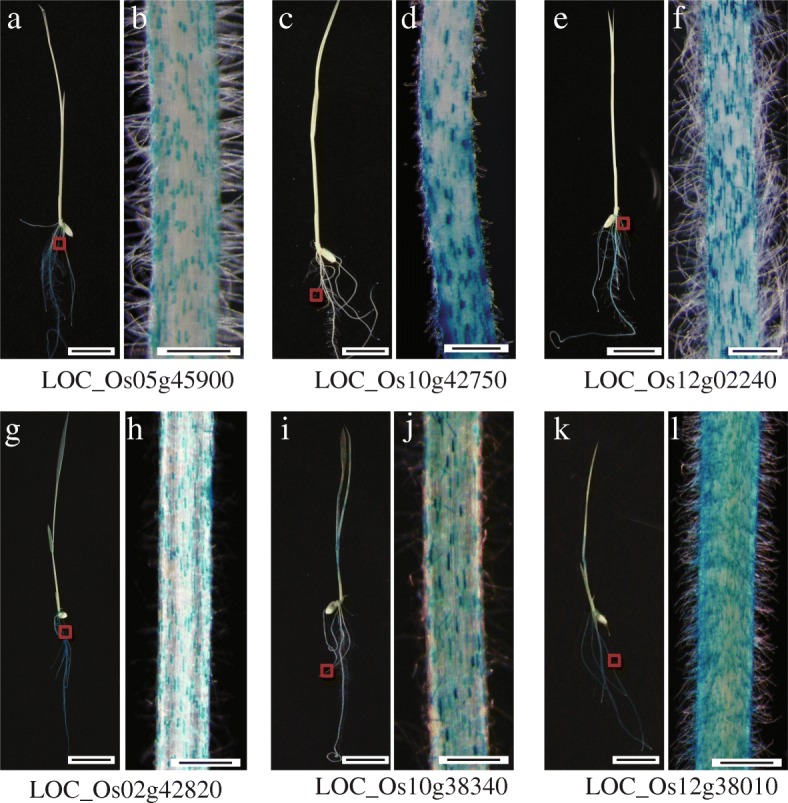
Validation of genes predominantly expressed in rice root hairs, using *GUS* reporter system. **a** to **f**, Expression patterns for reporter genes from promoter trap lines having T-DNA insertion within *LOC_Os05g45900* (**a** and **b**), *LOC_Os10g42750* (**c** and **d**), or *LOC_Os12g02240* (**e** and **f**). g to l, *GUS* expression in root hairs from transgenic plants harboring *LOC_Os02g42820* promoter::GUS construct (**g** and **h**), *LOC_Os10g38340* promoter::GUS construct (**i** and **j**), or *LOC_Os12g38010* promoter::GUS construct (**k** and **l**). Scale bar = 1 cm (**a**, **c**, **e**, **g**, **i**, and **k**) and 1 μm (**b**, **d**, **f**, **h**, **j**, and **l**)

To verify the expression patterns of our candidate genes, we used a promoter trap system. This technique involves T-DNA that carries the promoterless *GUS* reporter gene, as we have described previously (Jeon et al. 2000). We selected 63 lines with an in-frame fusion of the promoterless *GUS* in the genic region of 409 genes preferentially expressed in the root hairs. The patterns of GUS expression for those 63 promoter trap lines are presented in Additional file 1: Figure S2. From these, we identified three lines that exhibited root hair-preferential GUS staining patterns, as indicated in the microarray data and RT-PCR analysis (Fig. 2a-f). The T-DNAs were inserted into *LOC_Os05g45900*, encoding endonuclease (Fig. 2a and b); *LOC_Os10g42750*, encoding *OsCSLD1* (Fig. 2c and d); and *LOC_Os12g02240*, encoding hypothetical protein (Fig. 2e and f). The root hair-specific expression pattern of *OsCSLD1* was reported earlier (Kim et al. 2007). Co-segregations between the T-DNA insertion and GUS expression were checked, and root hair-preferential expression was detected via real-time PCR (Additional file 1: Figures S3 and S4).

In separate experiments, we generated transgenic plants harboring promoter and *GUS* fusion constructs. Three genes with expression values > 15 log_2_ in the root hairs were chosen: the promoters from *LOC_Os02g42820*, encoding TF L2 (Fig. 2g and h); *LOC_Os10g38340*, encoding gluthathione S-transferase (Fig. 2i and j); and *LOC_Os12g38010*, encoding methallothionein (Fig. 2k and l). All exhibited *GUS* expression in the root hairs. Although this preference was revealed by real-time PCR (Additional file 1: Figure S4), *GUS* activity was also observed in the shoots from transgenic plants, suggesting that the promoter region selected here was not sufficient to induce a root hair-preferential expression pattern (Fig. 2g, i and k).

### Gene ontology (GO) enrichment and MapMan analysis of genes preferentially expressed in rice root hairs

Using the enrichment tool in the rice oligonucleotide array database, we assigned 235 genes to 422 GO terms in Biological Processes (Additional file 2: Table S2). Thirteen GO terms were over-represented in genes preferentially expressed in root hairs (Table 1). The most enriched biological process was oxygen transport, as represented by non-symbiotic hemoglobin genes (31.25-fold enrichment value). Given the fact that the rate of growth for *Arabidopsis* root hairs is 1 μm min^− 1^, dynamic cell wall biosynthesis and structural alteration are essential for their development and formation (Griersona and Schiefelbein 2002). Accordingly, we noted that cell wall biogenesis (13.39), cellulose metabolic processes (5.10), and cell wall organization (2.84) were over-represented (Table 1). Genes involved in exocytosis were also up-regulated (5.52), possibly because they are involved in accelerating the rate of cell growth. Furthermore, genes related to auxin stimulus (8.15) and oxidative stress (6.55) were enriched in root hairs. Biological processes associated with the transport of chloride (6.25) or potassium (2.31) were enriched, confirming that one of the major functions of root hairs is nutrient uptake from the soil. The lipid catabolic process, represented by patatin-related phospholipase (5.86), and processes related to lipid transport (3.54), were also enriched in the root hairs. The GO terms for signal transduction (3.75) and protein amino acid phosphorylation (2.47) were also enriched in root hairs.

**Table 1:** Analysis of significantly enriched Gene Ontology terms for root hair-preferential genes from rice

GO Category	No. of GO repeats^a^	No. of GO repeats in queried genes^b^	No. of expected GO repeats^c^	Fold-enrichment value^d^
Oxygen transport	9	3	0.10	31.25
Cell wall biogenesis	21	3	0.22	13.39
Cellulose metabolic process	55	3	0.57	5.10
Cellular cell wall organization	132	4	1.41	2.84
Exocytosis	51	3	0.54	5.52
Response to auxin stimulus	46	4	0.49	8.15
Response to oxidative stress	186	13	1.98	6.55
Chloride transport	30	2	0.32	6.25
Potassium ion transport	203	5	2.16	2.31
Lipid catabolic process	48	3	0.51	5.86
Lipid transport	106	4	1.13	3.54
Signal transduction	200	8	2.13	3.75
Protein amino acid phosphorylation	1593	42	16.99	2.47

The rice genome contains 39,571 GO terms in all. A total of 409 genes preferentially expressed in root hairs were queried for GO term analysis. Similar GO categories are boxed

^a^number of selected GO Slim terms annotated in genome

^b^number of selected GO Slim terms observed in queried genes preferentially expressed in root hairs

^c^expected number of selected GO Slim terms in queried genes preferentially expressed in root hairs

^d^relative ratio of observed number to expected number for a selected GO Slim term

**Fig. 3:**
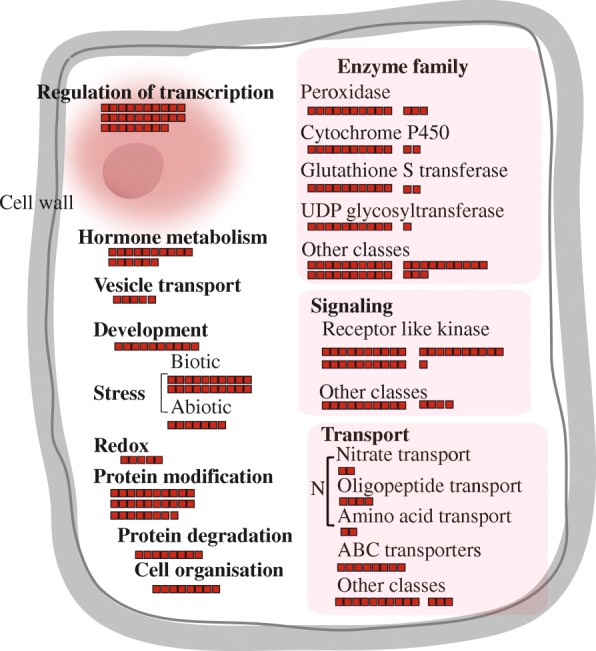
MapMan analysis of root hair-preferentially expressed genes. Cell Function overview analyzed with 409 genes. Enzyme family (81 genes), signaling transduction (45), and transport (29) were most abundant, as shown with red boxes. Small boxes in overview indicate root hair-specific genes. For enzyme families, peroxidases (13 genes), cytochrome P450 (12), glutathione S transferase (12), UDP glycosyltransferases (11), and other classes (33) were identified. Among 45 genes involved in signaling function, 31 loci encode receptor-like kinases and 14 are in other classes. For transport, 8 genes encode ABC transporters; 8 are involved in uptake of nitrogen sources such as nitrate, peptides, and amino acids; and 13 are in other classes. Detailed information about MapMan overview is shown in Additional file 2: Table S3

Our MapMan analysis (version 3.5.1R2) displayed a high-throughput dataset in diagrams for several diverse categories, e.g., metabolism, regulation, and cell functions (https://mapman.gabipd.org/) (Jung and An 2012). Among the various overviews installed in the MapMan toolkit, we primarily used the one for Cell Function. Based on this analysis, we found that genes in the enzyme family (81 genes), signaling transduction (45), and transport (29) were predominant (Fig. 3; Additional file 2: Table S3). In the enzyme family category, peroxidases (13 genes) were the most abundant. Cytochrome P450 (12 genes) and gluthathione S-transferase (12 genes) were also enriched in our rice samples. In accordance with previous reports in *Arabidopsis*, we also found an abundance of UDP glycosyl transferases (11 genes). Among the 45 genes involved in signaling functions, 31 loci encode receptor-like kinases. Among our 29 genes encoding transporters, eight are involved in the uptake of various nitrogen sources, e.g., nitrate, peptides, or amino acids.

### Promoter analysis of genes preferentially expressed in rice root hairs

Root hair-specific *cis*-elements (RHEs) have been found in several root-hair genes in *Arabidopsis* (Kim et al. 2006). The core RHE consists of 16 or 17 nucleotides. Here, we investigated the presence of RHE sequences in 2-kb promoter regions of our 409 root hair-preferentially expressed genes. From this, we identified 254 RHE sequences from the promoters of 177 candidate genes. The RHE sequences and positions within the promoter of each gene are presented in Additional file 2: Table S4.

To find additional consensus *cis*-regulatory elements (CREs) that induce root hair-preferential expression, we analyzed the 2-kb promoter regions of those 409 genes by using a locally installed MEME program (version 4.11.4) (Bailey et al. 2015). The top six motifs with higher E-values that were discovered here were then compared with the *Arabidopsis* DNA affinity purification motif database, using the Tomtom tool in MEME (Additional file 1: Figure S5) (O’Malley et al. 2016). Those six motifs showed similarity with the following CREs: binding site of basic pentacysteine1/BPC1, ethylene and salt responsive ERFs/ESE1, reduced vernalization 1/VRN1, reproductive meristem19/REM19, and transcription factor 3A/TF3A.

### Comparative analysis of root hair-preferential genes from rice and *Arabidopsis*

For our comparative analysis of root hair-preferentially expressed genes between rice and *Arabidopsis*, we downloaded *Arabidopsis* Affymetrix array data in the NCBI GEO database, which included root-hair array data for GSM943445 and GSM943446 (Becker et al. 2014). Meta-anatomical expression analysis presented 405 root hair-preferential genes in *Arabidopsis* (Additional file 1: Figures S6 and S7; Additional file 2: Table S5). Finally, we searched for orthologs between rice and *Arabidopsis* using the Inparanoid database (http://inparanoid.sbc.su.se/cgi-bin/index.cgi) (Ostlund et al. 2010). Among the 409 rice genes, 315 had *Arabidopsis* orthologs (Additional file 2: Table S6). Similarly, we identified 289 *Arabidopsis* genes with rice orthologs (Additional file 2: Table S7). Overall, the proportion of orthologous pairs was high, i.e., 77% of rice genes with *Arabidopsis* orthologs and 71% of *Arabidopsis* genes with rice orthologs. We also checked the conservation in expression patterns for these orthologs by comparing them with selected root hair-preferential genes. This revealed 50 rice genes with root hair-preferential *Arabidopsis* orthologs and 38 *Arabidopsis* genes with root hair-preferential rice orthologs (Additional file 2: Table S6 and S7). While examining this conservation, we considered the similarity of both their sequences and expression patterns. Subsequently, we identified relatively low functional conservation, with 38 (9.4%) of the 405 *Arabidopsis* genes and 50 (12.2%) of the 409 rice genes having orthologs with similar expression patterns*.* These results indicated that rice has adapted to water-logged conditions that are quite different from the environment that supports *Arabidopsis* growth, a situation that suggests a set of different genes are required for that adaptation process.

### Proposed model of root-hair development using predicted protein–protein interactions (PPIs) and integrated multi-omics data

**Fig. 4:**
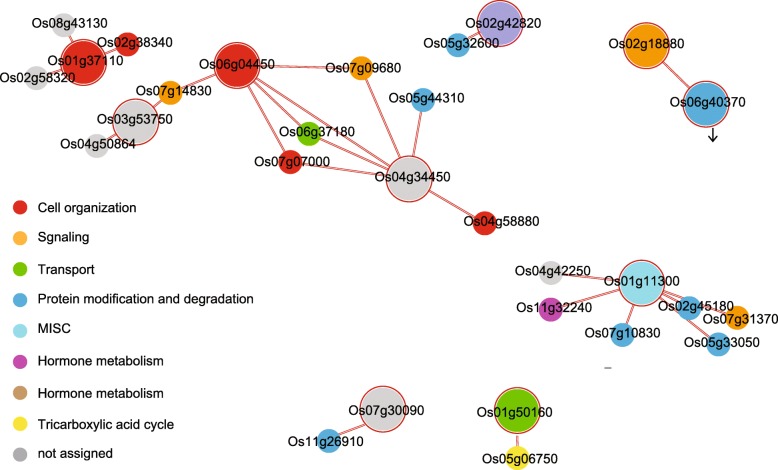
Proposed functional network of root hair-preferential genes. Networks were refined by showing nodes with PCC values > 0.5. Functional classifications using MapMan terms are incorporated in simplified networks, as indicated by colors within circles

To investigate the molecular network associated with root hair-preferential genes in rice, we analyzed the PPI network and coexpression. For the former, we uploaded 409 root hair-preferential genes to the rice interactions viewer (http://bar.utoronto.ca/interactions/cgi-bin/rice_interactions_viewer.cgi) and found 152 interactions associated with 29 root hair-preferential gene products (Additional file 1: Figure S8; Additional file 2: Table S8). Into this PPI network we then incorporated correlation coefficient values using Agilent 44 K anatomical meta-expression data that included root hairs. By doing so, we simplified the network to show the remaining nodes, which had Pearson correlation coefficient (PCC) values > 0.5 (Fig. 4). In addition, we combined functional classifications, using MapMan terms, with a simplified network that utilized different colors within the circles.

## Discussion

### Transcriptome of root hair-enriched samples

Root hairs play important roles in water and mineral uptake as well as interactions between plants and soil-borne bacteria. It is beneficial that we improve our understanding about how root hairs developed because we can then use that knowledge to generate plants with increased resistance under water- and mineral-deficit conditions. Extensive studies have been conducted during the past decade and high-throughput ‘omics’ databases are being constructed for *Arabidopsis* and soybean (Libault et al. 2010; Bruex et al. 2012; Lan et al. 2013). However, few data regarding root-hair development in rice are publicly available. Researchers suspect that different groups of genes and related molecular mechanisms might be required for that process because most cultivated rice is grown in paddy fields during the vegetative and early reproductive stages.

To search for relevant genes, we performed microarray analysis using RNAs isolated from root hair-enriched samples and examined the resultant data in silico. As an important advancement in biological research, microarrays have facilitated profiling of expression on the genomic scale. However, another technology, RNA sequencing (RNA-Seq), is rapidly replacing microarrays for large-scale studies. Although a strong correlation has been reported between gene expression profiles generated on the two platforms, RNA-Seq is more advantageous because it can be used to detect novel transcripts and genetic variants and its high sensitivity enables the detection of low-abundance transcripts as well as small RNAs. In contrast, it is more difficult to detect genes with low expression via microarrays because of background noise and cross-hybridization (Zhao et al. 2014). Thus, we cannot rule out the possibility that other root hair-specific genes with low expression levels, which were not detected in our microarray analysis, also contribute to the determination of root-hair specificity in rice.

We found 409 genes preferentially expressed in rice root hairs and confirmed expression patterns for six of them by applying the *GUS* reporter system. Screening potential promoter trap lines for those genes revealed that the efficiency of *GUS* expression was 4.8% (3/63), which is lower than the 9.09% (2/22) rate we have previously reported in pollen (Moon et al. 2018). Because we chose lines with T-DNA insertions within the root hair-preferential genes, the efficiency of *GUS* expression was much higher than we had observed. To explain this, we considered the following factors. Our vector included an intron with triple splicing donors/acceptors in front of the *GUS* reporter gene, which significantly enhanced the efficiency of *GUS* expression. However, we have previously found a splicing preference between the third donor and the first acceptor in our promoter trap lines (Kim et al. 2013). Therefore, we might conclude that two-thirds of these promoter trap candidate lines could not make functional GUS fusion protein. In addition, the threshold of *GUS* detection may have differed from that for microarrays. Occasionally, essential *cis*-acting elements may have been removed by the T-DNA insertion.

We identified RHEs in 2-kb promoter regions for 47% of the selected genes that were preferentially expressed in the root hairs. We believe that additional CREs for this preferential expression may occur in rice, based on our results from MEME analysis for promoters of other candidate genes (Additional file 2: Table S4 and Additional file 1: Figure S5). However, further examination is needed to elucidate the functional significance of those CREs.

### Transcriptome of root hairs provides useful clues about their development in rice

Through functional classification analysis of the root hair-preferential genes identified here, we found 13 GO terms and genes putatively involved in three different categories of Cell Function. In accordance with transcriptome data for root hairs from diverse species, expression levels were higher for rice genes participating in oxygen and mineral transport, auxin signaling, and antioxidant synthesis. Although no report has been made about any non-symbiotic hemoglobin gene specific to root hairs, constitutive expression of *Arabidopsis* class 1 hemoglobin leads to a reduction in root-hair development (Hunt et al. 2002). Because most rice cultivars are grown under water-logged conditions where oxygen levels are very low, those plants require an efficient system for oxygen uptake during their growth stages. Thus, it is conceivable that non-symbiotic hemoglobin genes that are highly expressed in root hairs can sequester oxygen in a hypoxic environment and provide an oxygen source to oxidate NADH for cell development (Sowa et al. 1998). The physiological role of auxin has been studied in detail and its involvement in root-hair development has been well established in *Arabidopsis* and rice. For example, overexpression of *AtTIR1*, an *Arabidopsis* auxin receptor, causes enhanced root hair growth and genetic mutations in several auxin-signaling components, which lead to defects in root-hair development (Wilson et al. 1990; Pitts et al. 1998). The mutation of a rice auxin influx transporter gene (*OsAUX1*) is linked to the production of shorter root hairs, and its phenotype is not rescued by exogenous indoleacetic acid (IAA) or 2,4-D treatments. Therefore, control of auxin levels by fine-tuning its signaling pathways is a determinant of proper root-hair development. The high expression of peroxidases in rice root hairs is likely related to the plant response to oxidative stress. Peroxidase decreases H_2_O_2_ levels but also catalyzes the production of reactive oxygen species (ROS) in the presence of strong reducing agents such as NAD (P) H, IAA, saturated fatty acids, or cysteine (Dunand et al. 2007; Csiszar et al. 2012). These ROS play an important role in root-hair development. For example, treating WT roots with a peroxidase inhibitor dramatically inhibits root-hair formation, and peroxidase-mediated ROS production is diminished in a root-hairless mutant of barley (Kwasniewski et al. 2013a). Two root-specific peroxidase genes, *HvPRX45* and *HvPRX2*, induce hydroxyl radicals in barley (Kwasniewski et al. 2013a). Moreover, *Arabidopsis* and the model legume *Lotus japonicus* contain three peroxidases specifically expressed in the root hairs: *RHS18*, *RHS19*, and *LjRH101* (Maekawa et al. 2005; Won et al. 2009). Taken together, results from our transcriptome analysis suggest that several, if not all, genes highly expressed in root hairs are essential for their formation and elongation. Therefore, this information provides useful insight into how root-hair development is controlled in rice.

### What is the functional similarity and diversity between rice and *Arabidopsis* genes preferentially expressed in root hairs?

For numerous plant species, their root hairs have the same purpose: uptake of water and nutrients. This common function might be supported by the high conservation of genes or proteins expressed in those structures. Although the proportion of orthologous pairs is high between rice and *Arabidopsis*, the conservation of expression patterns is low for root hair-preferentially expressed genes from those two species, which implies that each of them has evolutionarily developed unique features for adapting to different growth environments. This is consistent with a previous report that significant diversification in the structure and expression of a number of root hair specific genes was found among vascular plants (Huang et al. 2017). Many factors must be considered when attempting to explain this discrepancy. For example, we grew our rice seedlings on an MS medium to obtain root hairs, whereas the *Arabidopsis* samples were grown on a cellophane disc (Becker et al. 2014). Thus, we cannot ignore the effect that differences in culture methods and sample collection might have had on these results. From a physiological perspective, the hair cells and non-hair cells are of similar length during the hair-initiation stage in rice. Afterward, the hair cells elongate less than the non-hair cells (Kim and Dolan 2011), suggesting that some genes controlling such elongation are specifically expressed in those root hairs. In addition, dicot and monocot species differ in their cell wall structure and composition, including the level of fucogalactoxyloglucan, which might also explain the very low proportion of ortholog pairs for genes preferentially expressed in root hairs (Liu et al. 2015). The two species compared here utilize different types or amounts of nutrients. This is evidenced by rice roots, which have been adapted to a water-filled growing environment. Such an adaptation might partially explain the distinct contents of rice versus *Arabidopsis* genes. Because root hairs are the first site of organ contact with the soil, a diversity of genes preferentially expressed in root hairs might be necessary if plants are to adapt to new environments.

## Conclusion

Using Agilent 44 K microarray experiments and enriched root-hair samples, we identified 409 root hair-preferential genes. Functional classification of these candidates suggested that biological processes related to oxygen transport and cell wall generation are the most strongly associated with those genes. The expression patterns of six root hair-preferential genes were confirmed by using a *GUS* reporter system and quantitative RT-PCR analysis. These findings offer novel tools for future applications to enhance agronomic traits related to root hairs. Our results will be a useful reference to accelerate the understanding of the molecular mechanism for root-hair development in rice.

## Methods

### Plant materials and growth conditions

For isolation and morphological observation of root hairs, seeds of ‘Dongjin’ rice were sterilized with a 50% solution of sodium hydrochlorite for 30 min, and then washed three times in sterile distilled water. They were placed on an MSO medium containing 0.22% Murashige and Skoog (MS) basal salts and 1.2% Phyta agar (Duchefa) for 3 d at 27 °C to germinate.

### Root-hair isolation and RNA extraction

Seminal roots were detached from the 3 DAG seedlings and immediately submerged in liquid nitrogen. To detach the root-hair tissues, we gently rubbed each root surface with a brush. Four biological replicates were prepared. Total RNA was extracted with TRIzol and purified with an RNeasy plant mini kit (Qiagen). For synthesis of complementary DNA (cDNA), 1 mg of total RNA was reacted with Moloney murine leukemia virus reverse transcriptase (Promega), 2.5 mM deoxyribonucleotide triphosphate, and 10 ng of oligo (dT). To check for impurities caused by contamination of other tissues, we examined the expression of *OsBOR1* and *OsEXPA17*. Expression of *OsUbi5* was used as internal control. All primer sets are listed in Additional file 2: Table S9.

### Microarray experiments and collection of microarray data

Agilent microarrays (Agilent Technologies) were used to analyze the root hairs (Rice oligo microarray, 4 × 44 K). The cRNA was generated from RNA prepared separately from the four replicates of root-hair samples, following the manufacturer’s recommended protocols for the low-input quick amp labeling kit, one-color, (Agilent; 5190–2305). Afterward, 100 ng of the total RNA was transcribed to double-stranded cDNA and synthesized into complementary RNA (cRNA). Finally, labeled cRNA with cyanine-3-CTP was hybridized onto Rice Gene Expression Microarrays, 4x44K, containing 43,803 probes (Agilent; G2519F-015241). After washing, the arrays were scanned with an Agilent Surescan microarray scanner. Feature Extraction software (version 11.5.1.1; Agilent Technologies) was used to analyze the array images and obtain raw data. All microarray experiments and data-processing were performed in the system biology laboratory at Daegu Gyeongbuk Institute of Science and Technology.

Intensity values were initially normalized and log_2_-transformed as we described previously (Cao et al. 2012). The resultant data (GSE109811) were merged with Agilent microarray dataset GSE21494, downloaded from NCBI GEO. For comparative transcriptome analysis, we downloaded *Arabidopsis* Affymetrix microarray data series GSE5630, GSE5633, GSE5631**,** GSE5632, GSE5634, GSM943445, and GSM943446. The raw data files (.CEL files) corresponding to root-hair samples were then normalized by the affy package in R and converted into a log_2_ scale. These were used for KMC analysis, heatmap construction, and the identification of genes preferentially expressed in rice root hairs (Chandran et al. 2016).

### Plasmid construction and plant transformation

To generate transgenic plants with the *GUS* expression system under the control of root hair-specific genes, we chose *LOC_Os02g42820, LOC_Os10g38340*, and *LOC_Os12g38010*. To isolate the promoter regions of candidate genes, we performed PCR amplifications with gene-specific primer sets (Additional file 2: Table S9). The amplified DNA was inserted into the pGEM-T Easy Cloning Kit (Promega, Madison, WI, USA). Ligation products were used to obtain transformed colonies of *Escherichia coli* Top10, which were selected on LB plates supplemented with 50 μg mL^− 1^ ampicillin. Plasmid DNA was extracted with a plasmid DNA extraction kit (Geneall, Seoul, South Korea). Inserts were sequenced to select the correct clones. Following digestion with Hpa1 and Xba1 for *LOC_Os02g42820* (1404 bp), *LOC_Os10g38340* (1832 bp), and *LOC_Os12g38010* (2183 bp), the DNAs were ligated into the pGA3519 vector. After transformation into Top10, the plasmid DNAs were extracted and transferred into *Agrobacterium tumefaciens*. We used tissue culture techniques to obtain transgenic plants harboring the plasmids of interest.

### Histochemical GUS assay and microscopic analyses

Histochemical GUS-staining was performed for three promoter trap lines with T-DNA carrying promoterless *GUS* and three transgenic plants harboring the promoter::GUS construct. Seven-day-old seedlings were immersed in staining solution [100 mM sodium phosphate (pH 7), 5 mM potassium ferricyanide, 5 mM potassium ferrocyanide, 0.5% Triton X-100, 10 mM EDTA (pH 8), 0.1% 5-bromo-4-chloro-3-indolyl-β-d-GlcA/cyclohexylammonium salt, 2% dimethyl sulfoxide, and 5% methanol] and then vacuum-infiltrated. After the tissues were incubated at 37 °C for 6 h, the chlorophyll was removed in 70% EtOH. The assayed roots were photographed with a SZX61 microscope (Olympus, Tokyo, Japan). Genes inducing root hair-preferential *GUS* expression (*LOC_Os05g45900*, *LOC_Os10g42750*, and *LOC_Os12g02240*) exhibited an average of 13-, 14-, and 15-fold higher levels, respectively, of normalized expression on the microarray.

### Tests of gene ontology term enrichment and MapMan analysis

To examine GO enrichment within the Biological Processes category, we used the GO tool to query 409 genes preferentially expressed in root hairs (Cao et al. 2012). Significant terms in the GO category were selected if they had hypergeometric *p*-values ≤0.05 and at least two-fold enrichment values, as we had previously determined (Yoo et al. 2015). The MapMan program allows one to group genes into different functional categories and visualize data through various diagrams. To obtain their functional classifications, we uploaded the Rice Genome Annotation Project (RGAP) Locus IDs for 409 genes preferentially expressed in root hairs to the MapMan program (Usadel et al. 2009). We then investigated the Cell Function overview based on the diverse overviews installed in that kit.

### Network analysis

To develop a hypothetical functional network mediated by genes preferentially expressed in root hairs, we used the rice interactions viewer (Ho et al. 2012). The file for this network was uploaded from the rice interactions viewer to the CytoScape version 2.8.1 software (http://cytoscape.org) (Smoot et al. 2011). For whole genes within the network, we used the “File>Import>Attribute from Table” function (Additional file 1: Figure S6) to integrate root hair-preferential expression patterns in the red boundary nodes. Target genes were distinguished by nodes that were larger than the linked nodes and were marked with each locus number. Details for the RGAP locus_IDs and gene names used in the refined network are shown in Additional file 2: Table S8.

### Promoter analysis

To acquire the 2-kb promoter regions of 409 root hair-preferentially expressed genes, we used The Rice Annotation Project Database (http://rapdb.dna.affrc.go.jp/) (Sakai et al. 2013). The RHE sequences were searched in those 2-kb promoter regions by applying the Find Individual Motif Occurences tool from the MEME suite (http://meme-suite.org/tools/fimo) (Bailey et al. 2015). To identify any additional consensus CREs that induce root hair-preferential expression, we analyzed those promoter regions with the locally installed MEME program (version 4.11.4) from the MEME suite (Bailey et al. 2015). We then selected the top six motifs with higher E-values and compared them with the *Arabidopsis* DNA-affinity-purification motif database, using Tomtom in MEME (Additional file 1: Figure S5) (O’Malley et al. 2016).

### Ortholog detection

Protein sequences for rice and *Arabidopsis* were downloaded from RGAP (http://rice.plantbiology.msu.edu/) and The Arabidopsis Information Resource (https://www.arabidopsis.org/), respectively. For the ortholog search, we used the standalone version 4.1 of Inparanoid.

## Additional files

Additional file 1: **Figure S1.** Expression graph after KMC analysis of anatomical meta-expression data including root hairs. Genes in Cluster 7 (marked with red box) were selected as group showing root hair-preferential pattern of expression. **Figure S2.** *GUS* expression pattern of 63 promoter trap candidates. Among them, five lines showed *GUS* expression (blue and red boxes), and 3 exhibited root hair-preferential pattern (red box). **Figure S3.** Co-segregation between genotyping and *GUS* expression, as checked for 3 promoter trap lines: *LOC_Os05g45900* (a), *LOC_Os10g42750* (b), and *LOC_Os12g02240* (c). *GUS*-positive and *GUS*-negative are represented by + and -, respectively. **Figure S4.** Expression profiles of root hair-preferential genes. Analysis of expression patterns via real-time PCR for 6 genes: *LOC_Os05g45900* (a), *LOC_Os10g42750* (b), *LOC_Os12g02240* (c), *LOC_Os02g42820* (d), *LOC_Os10g38340* (e), and *LOC_Os12g38010* (f). Y-axis, gene expression relative to rice *OsUbi5* transcript level. **Figure S5.** New motifs discovered in 409 root hair-preferential genes, based on MEME analysis. **Figure S6.** Expression graph after KMC analysis of anatomical meta-expression data in *Arabidopsis*. Clusters 2, 4, and 6 (in red boxes) show root hair-preferential expression patterns. **Figure S7.** Heatmap for expression profiles of root hair-preferential genes in *Arabidopsis*. Yellow, high expression; dark-blue, low expression. **Figure S8.** Functional gene network associated with root hair-preferential genes in rice, as indicated by large circles with red boundaries. Six functionally characterized genes are named. Interactions between nodes with PCC values > 0.5 are represented by red lines. (DOCX 2500 kb)

Additional file 2: **Table S1.** Selected rice root hair-preferentially expressed genes. **Table S2.** Classification of GO terms for Biological Processes associated with rice root hair-preferential genes. **Table S3.** MapMan classification of root hair-preferential genes in rice. **Table S4**. RHE sequences and positions within promoters of root hair-preferentially expressed genes. **Table S5.** Locus numbers and putative functions of root hair-preferential genes of *Arabidopsis*. **Table S6.** *Arabidopsis* orthologs for rice root hair-preferential genes. **Table S7.** Rice orthologs for *Arabidopsis* root hair-preferential genes. **Table S8.** Predicted protein–protein interactions of root hair-preferential genes. **Table S9.** Primer sequences used in this study. (XLSX 884 kb)

## Abbreviations

cDNA: Complementary DNA
GO: Gene Ontology
IAA: Indoleacetic acid
KMC: K-means clustering
PCC: Pearson correlation coefficient
PPI: Protein–protein interaction
RGAP: Rice Genome Annotation Project
WT: Wild type
