# Supplementary material for: Genome-wide analysis of root hair-preferential genes in rice
Source: Rice (N Y). 2018 Aug 29;11:48. doi: 10.1186/s12284-018-0241-2 (PMC6115326; doi:10.1186/s12284-018-0241-2)
Supplement: Supplementary file 1 — Figure S1. Expression graph after KMC analysis of anatomical meta-expression data including root hairs. Genes in Cluster 7 (marked with red box) were selected as group showing root hair-preferential pattern of expression. Figure S2. GUS expression pattern of 63 promoter trap candidates. Among them, five lines showed GUS expression (blue and red boxes), and 3 exhibited root hair-preferential pattern (red box). Figure S3. Co-segregation between genotyping and GUS expression, as checked for 3 promoter trap lines: LOC_Os05g45900 (a), LOC_Os10g42750 (b), and LOC_Os12g02240 (c). GUS-positive and GUS-negative are represented by + and -, respectively. Figure S4. Expression profiles of root hair-preferential genes. Analysis of expression patterns via real-time PCR for 6 genes: LOC_Os05g45900 (a), LOC_Os10g42750 (b), LOC_Os12g02240 (c), LOC_Os02g42820 (d), LOC_Os10g38340 (e), and LOC_Os12g38010 (f). Y-axis, gene expression relative to rice OsUbi5 transcript level. Figure S5. New motifs discovered in 409 root hair-preferential genes, based on MEME analysis. Figure S6. Expression graph after KMC analysis of anatomical meta-expression data in Arabidopsis. Clusters 2, 4, and 6 (in red boxes) show root hair-preferential expression patterns. Figure S7. Heatmap for expression profiles of root hair-preferential genes in Arabidopsis. Yellow, high expression; dark-blue, low expression. Figure S8. Functional gene network associated with root hair-preferential genes in rice, as indicated by large circles with red boundaries. Six functionally characterized genes are named. Interactions between nodes with PCC values > 0.5 are represented by red lines. (DOCX 2500 kb) [file 12284_2018_241_MOESM1_ESM.docx]

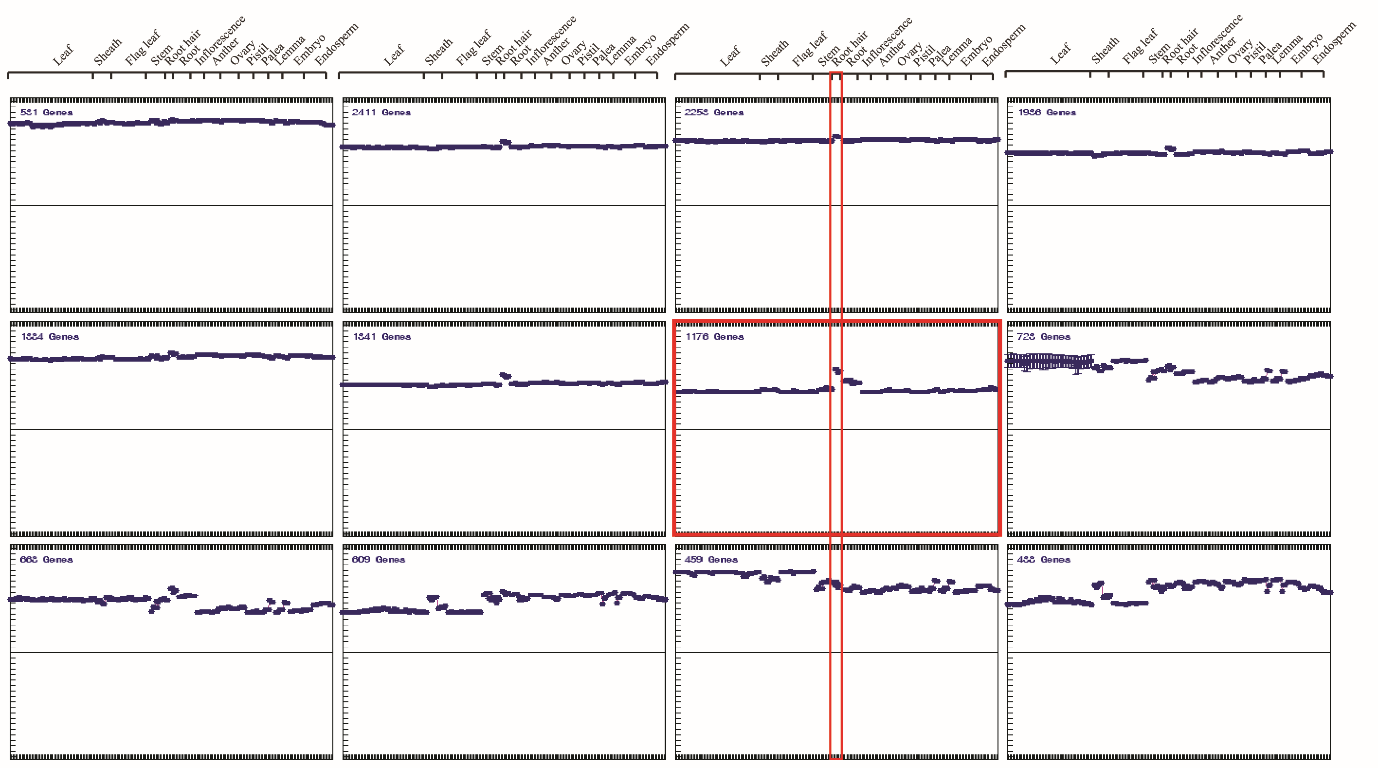


**Figure S1**. Expression graph after KMC analysis of anatomical meta-expression data including root hairs. Genes in Cluster 7 (marked with red box) were selected as group showing root hair-preferential pattern of expression.


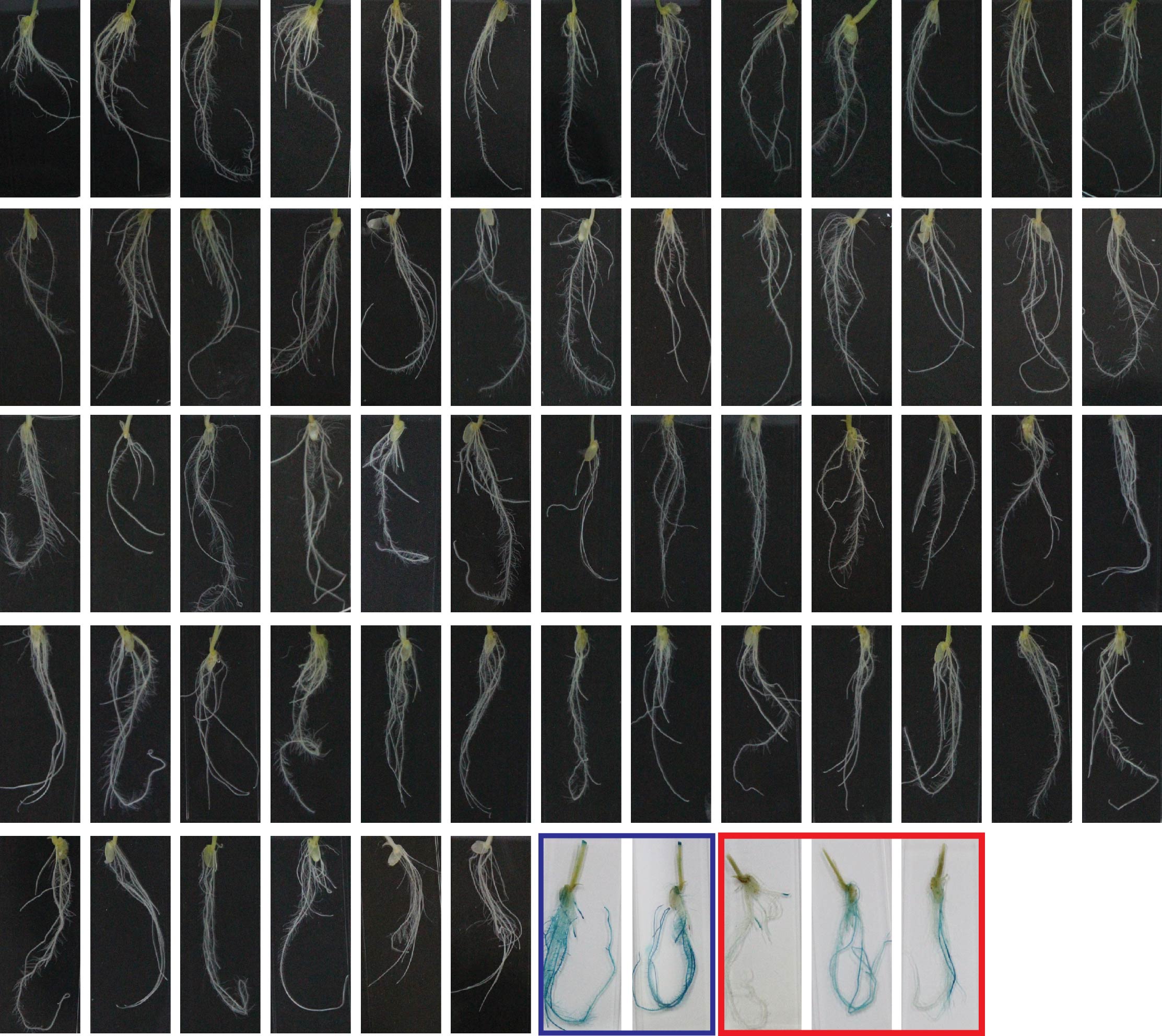


**Figure S2**. *GUS* expression pattern of 63 promoter trap candidates. Among them, five lines showed *GUS* expression (blue and red boxes), and 3 exhibited root hair-preferential pattern (red box).


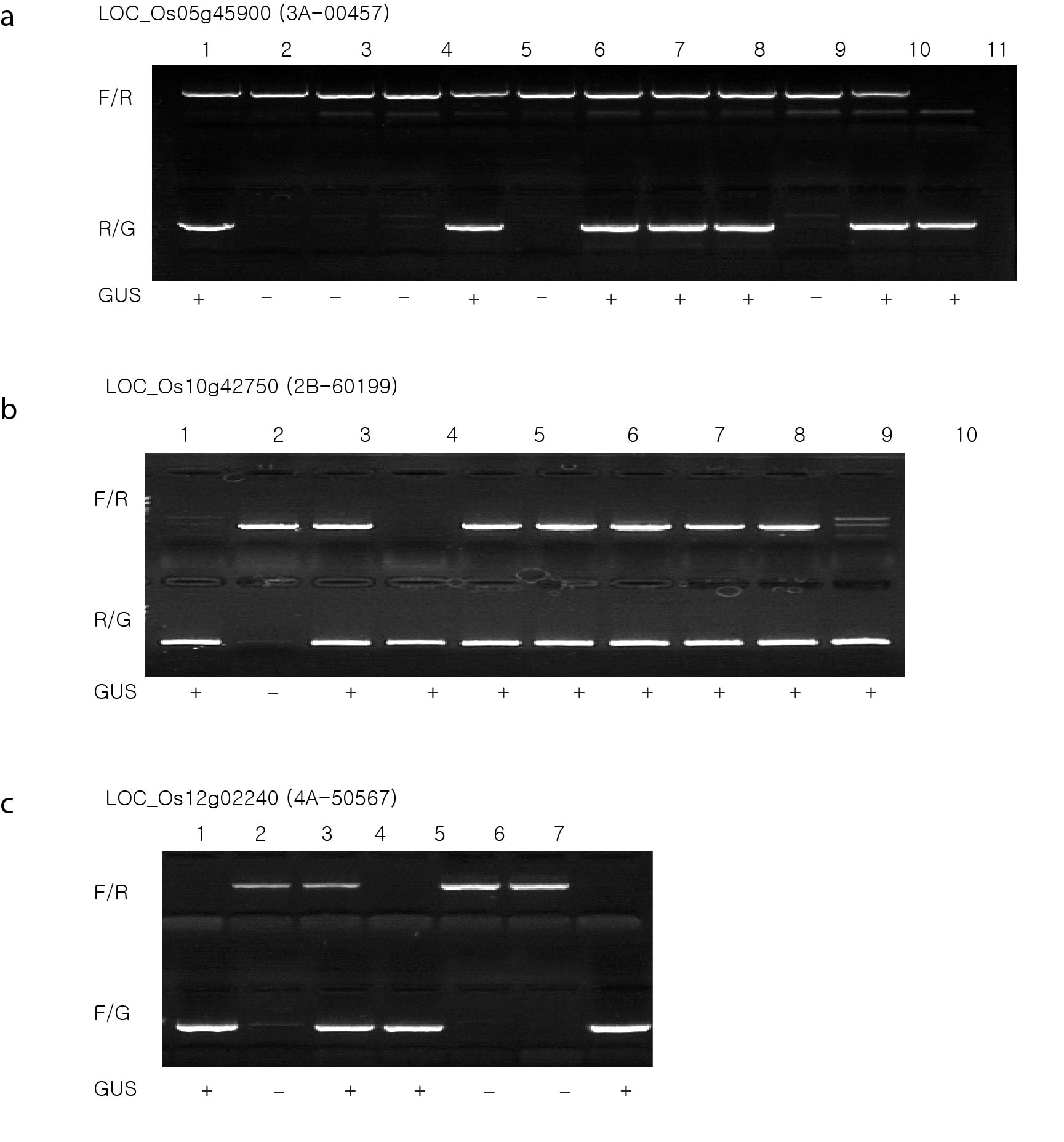


**Figure S3**. Co-segregation between genotyping and *GUS* expression, as checked for 3 promoter trap lines: *LOC_Os05g45900* (a), *LOC_Os10g42750* (b), and *LOC_Os12g02240* (c). *GUS*-positive and *GUS*-negative are represented by + and -, respectively.


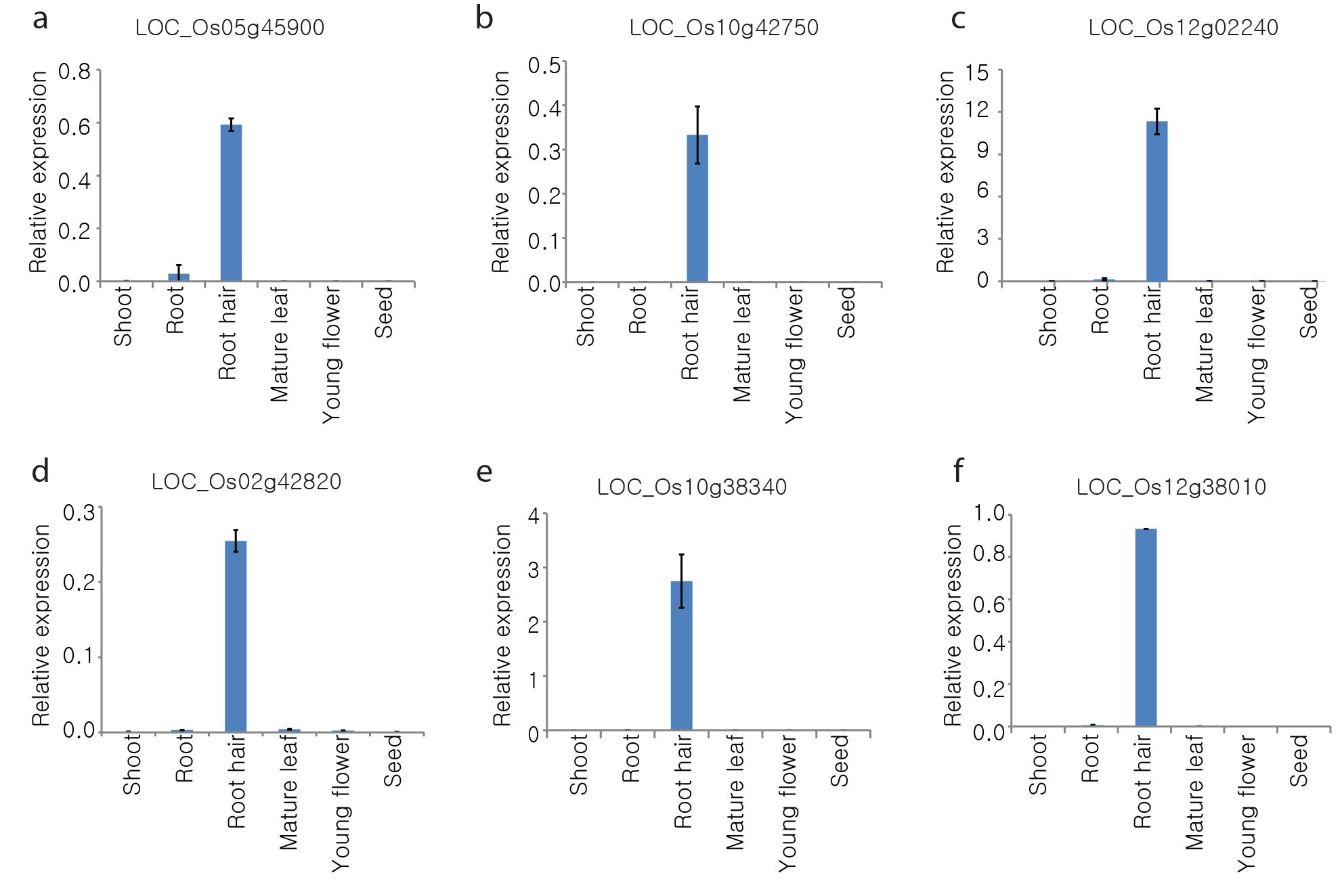


**Figure S4**. Expression profiles of root hair-preferential genes. Analysis of expression patterns via real-time PCR for 6 genes: *LOC_Os05g45900* (a), *LOC_Os10g42750* (b), *LOC_Os12g02240* (c), *LOC_Os02g42820* (d), *LOC_Os10g38340* (e), and *LOC_Os12g38010* (f). Y-axis, gene expression relative to rice *OsUbi5* transcript level.


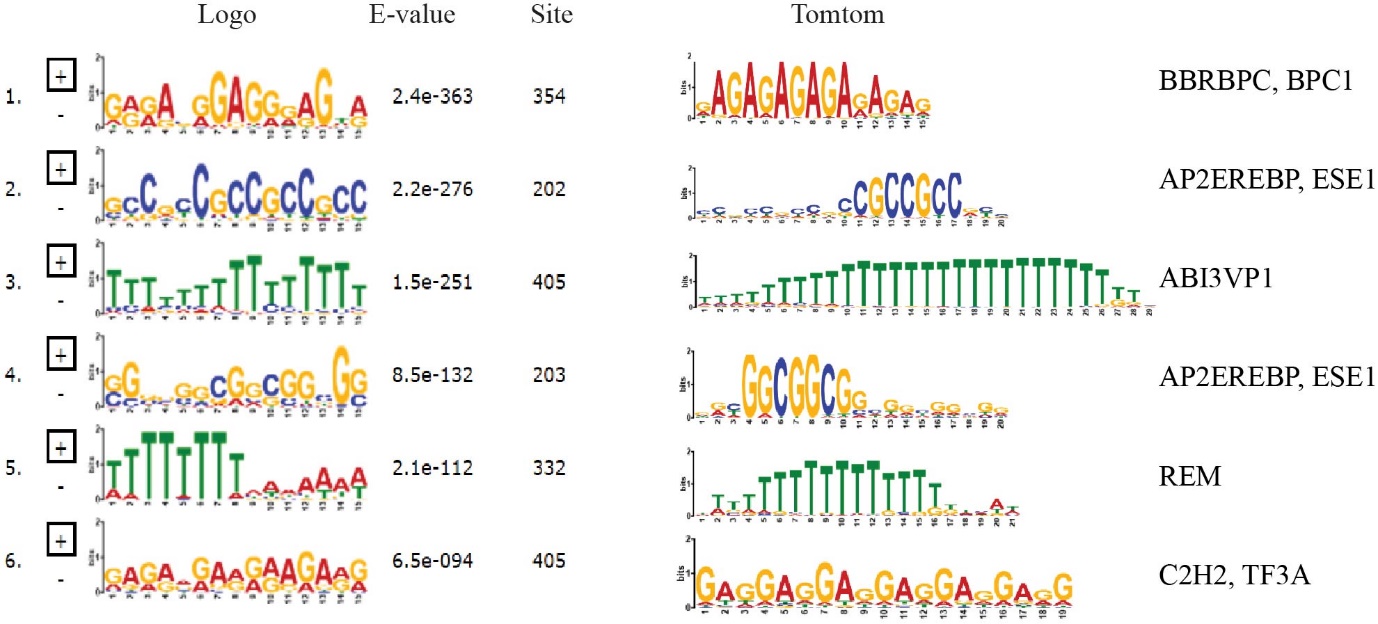


**Figure S5**. New motifs discovered in 409 root hair-preferential genes, based on MEME analysis.


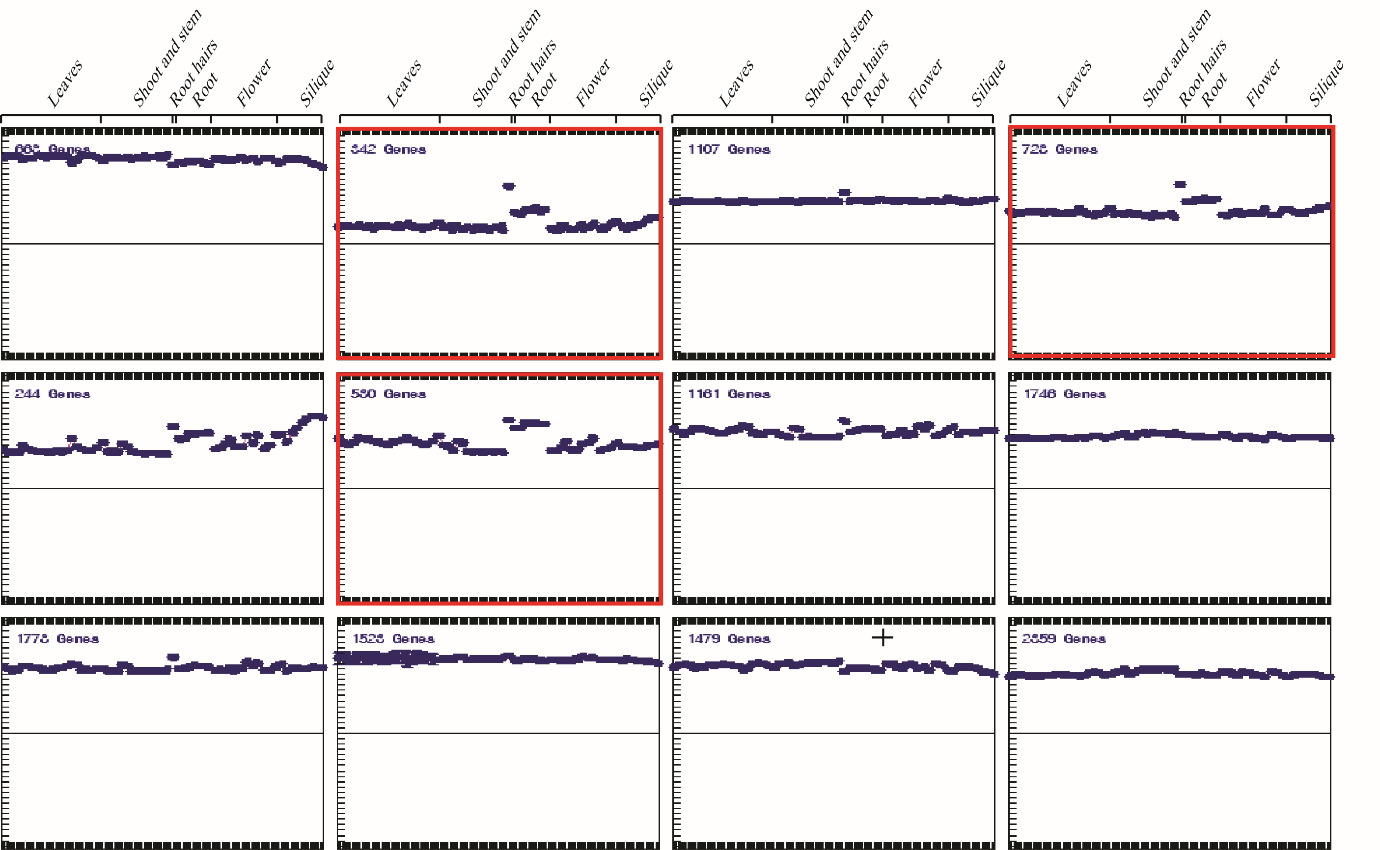


**Figure S6**. Expression graph after KMC analysis of anatomical meta-expression data in *Arabidopsis*. Clusters 2, 4, and 6 (in red boxes) show root hair-preferential expression patterns.


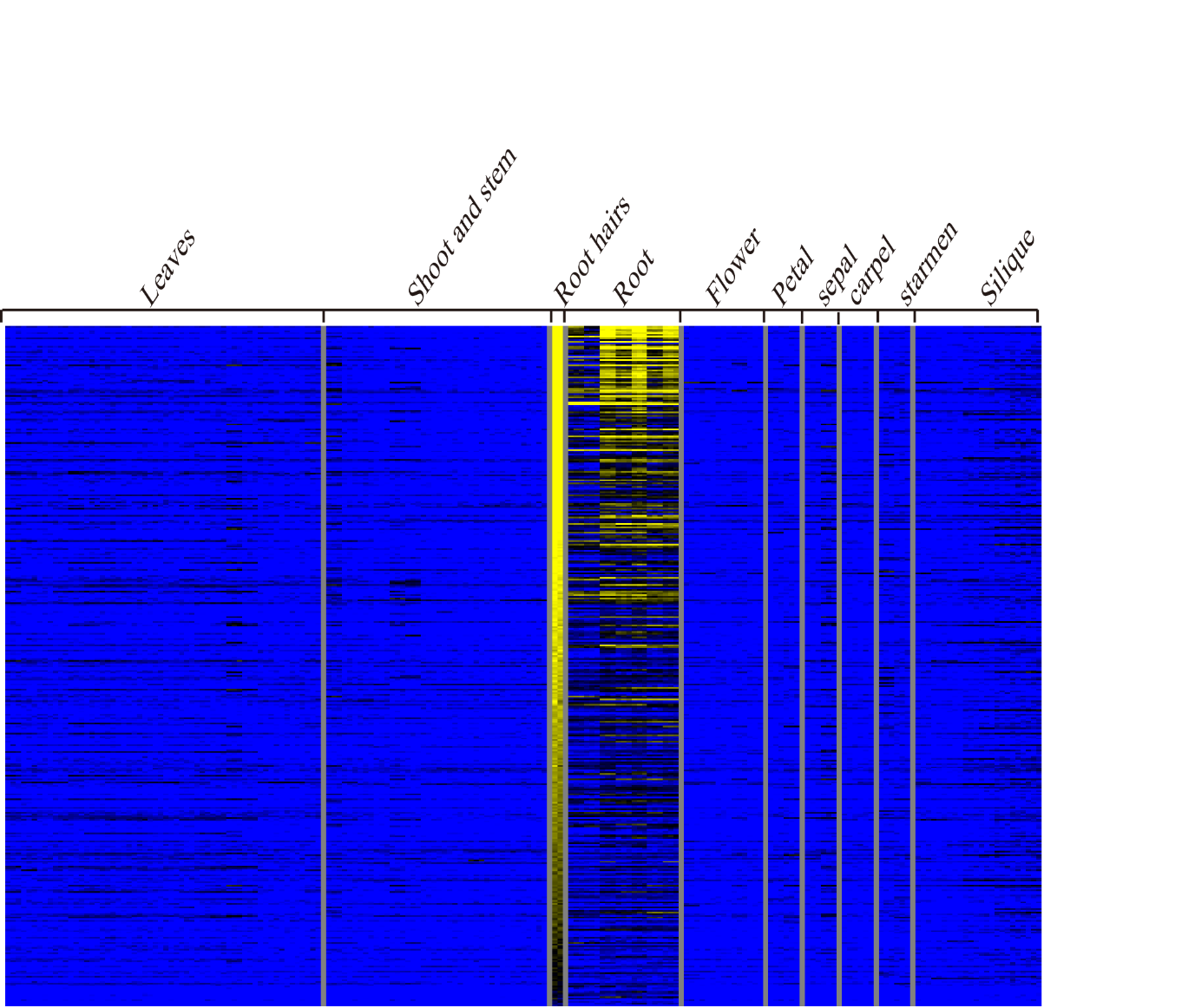


**Figure S7**. Heatmap for expression profiles of root hair-preferential genes in *Arabidopsis*. Yellow, high expression; dark-blue, low expression.


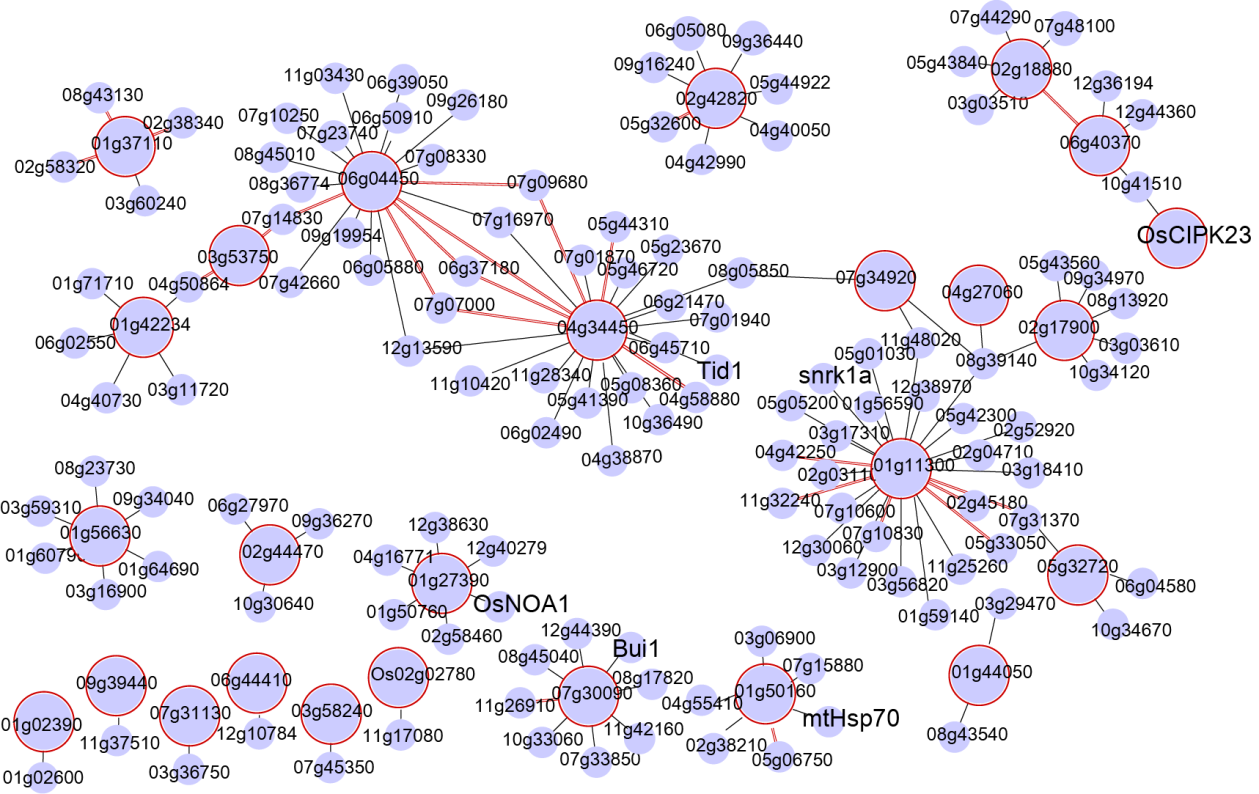


**Figure S8**. Functional gene network associated with root hair-preferential genes in rice, as indicated by large circles with red boundaries. Six functionally characterized genes are named. Interactions between nodes with PCC values >0.5 are represented by red lines.
